# A pathological and clinical study of 706 primary tumours of the ovary in the largest tertiary hospital in Ghana

**DOI:** 10.1186/s12905-017-0389-8

**Published:** 2017-04-17

**Authors:** Patrick Kafui Akakpo, Leonard Derkyi-Kwarteng, Richard Kwasi Gyasi, Solomom Edward Quayson, Simon Naporo, Jehoram Tei Anim

**Affiliations:** 1 Department of Pathology, School of Medical Sciences, University of Cape Coast, Cape Coast Teaching Hospital, Cape Coast, Ghana; 2 Department of Pathology, College of Health Sciences, University of Ghana Medical School Korle-Bu Teaching Hospital, Accra, Ghana

**Keywords:** Ovary, Tumours, Clinical, Histopathological, Features, Epidemiology, Ghana

## Abstract

**Background:**

Ovarian tumours are a leading cause of death in Ghana. Even though geographical and racial differences exist in the frequency, types and age distribution of primary ovarian tumours, information about the clinical and pathological characteristics of ovarian tumours in Ghana and its neighboring countries is scanty. We determined the frequency, age distribution, histopathological types and clinical features of primary ovarian tumours diagnosed at the Korle-Bu Teaching Hospital in Ghana to aid in the management of patients.

**Method:**

All pathology records of ovarian tumours diagnosed from January 2001 to December 2010 were reviewed. Histopathologically, tumours were classified according to the then World Health Organization 1999 classification. Biographical and clinical data of patients were also collected and entered into Epi-info to determine the frequency, age distribution and other clinical features of the types of ovarian tumour.

**Results:**

Seven hundred and six ovarian tumours were studied. Germ cell tumours were the most common (41.9%), with mean age of occurrence being 30.7 years (SD 12.7), they were dominated by mature teratomas (39.2%). Surface epithelial tumours were second, and commonly occurred in women aged 35–44years, 77 (26.8%). Sex cord stromal tumours followed with mean age of occurrence of 40.2 years (SD 17.9). The most common malignant tumours were surface epithelial (52.1%) dominated by serous carcinomas with mean age 50.1 years. Most patients (47.7%) presented within 1 month of onset of symptoms, feeling a lower abdominal mass (38.5%).

**Conclusion:**

The most common primary ovarian tumours in this study are Germ cell tumours, dominated by mature teratomas. Adenocarcinomas are mostly serous and occur in younger women compared to findings of other Western studies. The single most common malignant ovarian tumour in children and adolescents is Burkitt lymphoma. Patients who develop ovarian tumours have no specific symptoms or signs at presentation, to aid early diagnosis.

## Background

Malignant ovarian tumours are a leading cause of cancer death in Ghana [1, 2]. Ovarian cancer is ranked within the top five (5) causes of cancer mortality in Ghana [1, 2]. The mean age of occurrence of ovarian tumours is quoted as 46.4 years with most patients presenting with late stage disease [2]. In line with this, most deaths from ovarian cancer occur in women older than 34 years [1]. Similar findings have been reported in studies from Nigeria [2]. The burden of ovarian tumours as a whole is not known in Ghana.

There is a progressive increase in the incidence of ovarian tumour from the third decade until it peaks in the seventh decade [3, 4]. Different types of ovarian neoplasms predominate in different age groups and specific tumours may also occur, more frequently in specific races and in specific geographic locations of the world. There is however no data on the types, age distribution and presentation of ovarian tumours in Ghana. A study carried out in South Africa showed that germ cell tumours predominated in Blacks (52%), as opposed to Indians (19%) [5]. In Nigeria though the general trend is similar, there have been varying reports regarding the commonest primary malignant tumour of the ovary. While some studies report serous cystadenocarcinoma as the commonest malignant tumour others report granulosa cell tumours as the commonest. However in all these studies their sample size was less than 500 cases [6–8]. Again certain tumours are known to be endemic in certain regions, Burkitt lymphoma being an example. Literature on this is however sporadic and based on a few cases reported in Nigeria and Togo with no data on the situation in Ghana [9, 10].

Our aim was to determine the pathological types and features of ovarian tumours, the clinical characteristics of patients such as; age distribution, the duration and nature of presenting signs and symptoms, size of tumours and laterality of tumours. The study was carried out at the Korle-Bu Teaching Hospital, Ghana over a 10 year period when the hospital had the only functional pathology center in the country. The frequency, clinical and pathological features are compared with those of tumours in other races and countries and the similarities and differences discussed. Knowledge of the clinical characteristics, frequency and distribution of the types of ovarian tumours will aid clinicians in the management of patients with ovarian tumours. It will also inform the adoption of basic management protocols relevant to management of such tumours in our setting.

## Methods

A retrospective, descriptive study was conducted to determine the frequency and age distribution of various histopathological types of ovarian tumours. Clinical characteristics of patients such as the presenting signs and symptoms and their duration were studied. Histopathological features of tumours such as size and laterality were documented. These biographic data were collected from request forms and histopathological reports of all ovarian tumours diagnosed at the department of pathology of the Korle-Bu Teaching Hospital (KBTH) from January 2001 to December 2010. All histopathology records of these cases were reviewed by two pathologists. The tumours were classified at the time according to the WHO’s existing 1999 classification of ovarian tumours and thus our findings may be based on some existing misclassification of ovarian histology at that time. All cases without records were excluded from the study. Only primary ovarian tumours were studied.

### Data collection

Data were captured using the Epi Info™ Version 3.5.1 software. First in make view mode a structured data capturing page was developed to allow entry of the desired data. After all relevant data had been captured, the Analysis module was used to view, read and analyse data entered. Variables such as frequency, median and mean were derived for the various types of ovarian tumour. In addition other statistical variables were derived from the analysis mode. Statistical methods such as standard deviation and Chi squared comparisons to determine statistical significance were used.

## Results

In all, 706 ovarian tumours were analyzed. Overall, the most frequently occurring tumours were germ cell tumours, accounting for 296 (41.9%) of the tumours. These were followed by surface epithelial tumours, 287 (40.7%), then sex-cord stromal tumours 107 (15.2%) (Table 1). Surface epithelial tumours most commonly affected the 35–44years age group 77 (26.8%) cases (Table 2). Their distribution and behaviour are shown in Table 2. There were 100 patients with malignant surface epithelial tumours most (71) serous in type (Table 2). The mean age for serous carcinoma was 50.1 years (SD 10.0). The median age was 50.0 years. The mean age for mucinous carcinoma was 47.3 years (SD 7.1) and the median age was 45 years. The details are shown in Table 2. All the 71 serous carcinomas were less than 31 cm in largest diameter, with 35 (49.3%) being 0–10 cm in size. This was followed by 33 (46.5%) in the 11–20 cm range. Only 3 (4.2%) were larger than 20 cm. The mucinous carcinomas showed a similar size distribution with majority 8 (88.9%) being less than 20 cm. However, unlike the serous carcinomas 5 (55.6%) of the mucinous carcinomas ranged in sizes 11–20 cm compared to 46.5% of the serous carcinomas. Although they are not comparable in numbers, on average, the mucinous carcinomas were larger with a test of significance of; *χ* ^2^ (1, *n* = 80) =0.845, *p* < 0.05. Only 2 (5.1%) out of 39 bilateral malignant surface epithelial tumours were mucinous carcinomas. 34 were serous carcinomas. Mature teratoma was the most common germ cell tumour seen in this study, accounting for 277 (93.6%) of all germ cell tumours (Table 3) and most common between 25–34years (35%) mean age 31 years. It was also the most common ovarian tumour in this study. Mature teratoma was followed by the malignant germ cell tumours, the commonest of which was dysgerminoma 8 (2.7%), followed in order by, yolk sac tumours 5 (1.7%), immature teratomas 4 (1.4%), and one each (0.3%) of choriocarcinoma and monodermal teratoma (Table 3). The mean age of occurrence of germ cell tumours was 30.7 years (SD 12.7) with a median age of 29.5 years (Table 3). 19 of the germ cell tumours were bilateral, (including 16 mature teratomas, 2 dysgerminomas and 1 choriocarcinoma). There were 107 sex cord stromal tumours most (43%) being adult granulosa cell tumours (Table 3). The mean age of occurrence of sex cord stromal tumours was 40.2 years (SD 17.9). The mean age of occurrence of the 46 adult granulosa cell tumours, the most common sex cord-stromal tumour was 46.5 years (SD15.9) (Table 3). Only 6 of the sex cord stromal tumours were bilateral.

**Table 1:** Broad histopathological types of ovarian tumours

Ovarian tumour	Frequency	Percent
Germ cell	296	41.9
Surface epithelial	287	40.7
Sex cord stromal	107	15.2
Metastatic	8	1.1
Lymphoma	8	1.1
Total	706	100.0

**Table 2:** Age distribution, types and behaviour of surface epithelial tumours

	Age range in years							Tumour behaviour		
Type of tumour	15–24	25–34	35–44	45–54	55–64	>64	Total	Benign	Borderline	Invasive
Serous	13	42	49	50	31	11	196	112	13	71
Mucinous	7	13	19	9	9	3	60	41	10	9
Endometrioid	0	0	3	3	3	0	9	0	0	9
Undifferentiated	1	0	2	5	0	1	9	0	0	9
Brenner	0	1	2	3	1	0	7	6	0	1
Mixed	1	0	1	2	0	1	5	5	0	0
Clear cell	0	0	1	0	0	0	1	0	0	1
Total	22	56	77	72	44	16	287	164	23	100

**Table 3:** Age distribution of the various types of germ cell and sex cord–stromal tumours

	Age range in years								
Type of germ cell tumour	0–4	5–14	15–24	25–34	35–44	45–54	55–64	>64	Total
Mature teratoma	4	18	58	99	66	21	9	2	277
Dysgerminoma and variants	0	1	6	1	0	0	0	0	8
Yolk sac tumours	0	0	4	0	1	0	0	0	5
Immature teratoma	0	2	0	1	0	0	1	0	4
Choriocarcinoma	0	0	0	0	0	0	0	1	1
Monodermal teratoma	0	0	0	0	0	1	0	0	1
Total	4	21	68	101	67	22	10	3	296
Type of sex cord stromal tumour	0–4	5–14	15–24	25–34	35–44	45–54	55–64	>64	Total
Adult GCT ^a^	0	0	4	10	3	15	6	8	46
Fibroma	0	0	6	11	3	3	4	2	29
Sertoli-Leydig cell tumour, ID ^b^	0	0	6	0	2	2	0	0	10
Juvenile GCT	0	4	1	3	0	0	0	0	8
Thecoma- fibroma	0	0	1	0	2	3	0	2	8
Thecoma	0	0	0	0	1	2	0	1	4
Unclassified	0	0	1	0	0	0	0	1	2
Total	0	4	19	24	11	25	10	14	107

^a^ Granulosa Cell Tumour

^b^ Intermediate Differentiation

Regarding symptoms 38.5% complained of a lower abdominal mass, 18.3% of sudden abdominal pain that needed surgical attention, 17.4% of chronic lower abdominal pain, 17.4% of distension, 3.1% of menstrual disorders, and 0.4% of ‘virilizing’ signs. There were 4.9% of patients who had other ‘non-specific’ symptoms such as malaise, weight loss and easy fatigability. The commonest presenting sign was non tender lower abdominal mass in 41.1% patients, while 37.2% had a tender abdominal mass. Ascites was present in 10.6% of patients. In 10.6% of patients the ovarian tumours were found incidentally. Statistically there was no likelihood of any of the presenting signs presenting more often. Out of those who had duration of illness stated, 47.7% had symptoms for less than 1 month prior to presentation, while 42.4% had symptoms for between 1 month and 1 year. 9.3% had symptoms lasting more than 1 year and only one (0.6%) patient had symptoms for more than 5 years. Statistically there was no increased likelihood of a particular patient with a particular type of tumour presenting within a stated duration. *χ* ^2^ (3, *n* = 172) = 28.57, *p* < 0.05.

## Discussion

The most common tumours in this study were germ cell tumours (41.9%), at variance with a study in eastern India in which germ cell tumours formed the second major group of tumours (23.1%) [11]. Unlike blacks, the most common ovarian tumour in whites has been reported to be surface epithelial tumours [4, 11]. The proportion of germ cell tumours varied in other studies between 20 and 42.2% [11, 12]. However, in support of our findings, a significantly higher number of germ cell tumours have been reported from Lagos, Nigeria and from South Africa among blacks, [5, 13]. In the Nigerian study, tumours of germ cell origin accounted for 52.7% of ovarian neoplasms while surface epithelial tumours constituted 27.6% of 486 cases studied [13]. The South African study showed a predominance of germ cell tumours in Blacks, 52% (closer to what was found in the present study) as opposed to 19% in Indians [5]. Mature teratoma was overall, the most common ovarian tumour (39.2%) in this study. It made up 93.6% of germ cell tumours. The proportion of mature teratoma in the present study is much higher, compared to 15.9% of all ovarian tumours seen in the Eastern Indian study [11]. Dysgerminoma was the most common malignant germ cell tumour in this study (44.4%) similar to studies in the United States of America (56%) and India (36.2%) [11, 12]. Overall, it constituted 1.1% of all ovarian tumours, similar to the 1–2% reported in studies from the United States of America [9]. The large proportion of germ cell tumours is accounted for by the high proportion of benign mature cystic teratomas.

56.8% of ovarian surface epithelial tumours were benign while 35.2% were malignant and 23 (8.0%) borderline similar to findings in Eastern India and In the United States of America [11, 12]. In the present study, the greatest proportion of cancers is contributed by surface epithelial cancers, not different from the Indian study [9]. Ovarian serous tumours made up the largest proportion (68.7%) of epithelial tumours, similar to findings in the United States of America, Eastern India and Europe [11, 12]. In this study, however, they formed a smaller proportion of all malignant ovarian tumours (35.6%) compared to 50% in studies from the United States [9]. This may be due to geographical variations in the types of malignant ovarian tumours with malignant germ cell and malignant sex cord-stromal tumours contributing more significantly to the malignant tumours. Mucinous epithelial tumours of all kinds constituted 8.5% of all the ovarian tumours, about half the proportion found in other studies, in which it formed about 15% of ovarian neoplasms [9]. Again they formed 21% of all epithelial tumours. Invasive mucinous carcinomas formed 4.7% of all primary ovarian cancers and again 3.1% of primary epithelial tumours of the ovary. This is in line with studies elsewhere that suggest primary mucinous carcinomas of the ovary are not as common as previously thought (forming 3–4% of epithelial ovarian tumours) and that most are metastatic [9–11]. Regarding laterality, our findings agree with findings in other studies which concluded that mucinous carcinomas tend to be unilateral compared to serous carcinomas and that bilateral mucinous carcinomas tend to be metastatic [9, 10]. Some studies have shown that when optimized, an algorithm using tumor size and laterality (bilateral tumors of any size, or unilateral tumor <13 cm are likely metastatic; while unilateral tumor > or =13 cm are likely primary) can accurately classify majority of mucinous carcinomas [14, 15]. Endometrioid ovarian carcinoma is said to comprise 10–25% of all primary ovarian carcinomas [9]. It however formed only 4.5% of all malignant tumours of the ovary in our study, similar to findings in studies in Eastern India (4.2%) and Western India (5%) [11].

Sex cord-stromal tumours comprised 15.1% of all the ovarian tumours in this study, not too different from the 5–12% of all ovarian neoplasms reported in other studies [13, 16, 17]. They however, form a larger proportion (32.3%) of malignant ovarian tumours in this study, when compared to the 7% of all malignant ovarian tumours reported elsewhere [13, 16, 18]. The commonest sex cord-stromal (50.5%) tumour was granulosa cell tumour, similar to findings in other studies. Granulosa cell tumours form 27.3% of all malignant ovarian tumours reported in this study, significantly more than that reported in other studies [18]. Adult granulosa cell tumours constituted 85.2% of all the granulosa cell tumours, a finding that is close to the 95% reported in other studies [18]. These differences may again be due to geographical location and race.

The mean age of patients with malignant ovarian tumours was 49 years, older than the mean age of 45 years reported earlier from the same hospital [19]. Majority (94%) of the cancers occurred in women older than 35 years with most (34.3%) of them in the age ranges 45–54years, these were followed by those in the age ranges 35–44 years (27.5%) and then 55–64 years (24.3%). The affected age ranges are comparable to age ranges reported in India where most malignant tumours were found in women 41–60 years old [11]. Again the lower age at diagnosis is comparable to that reported for African- Americans with more cancers diagnosed in premenopausal African- American women compared to Whites in who malignant surface epithelial tumours mostly occur in the 60–65 year old group [3, 4], thus, ovarian cancer occurs in relatively younger women in our setting.

The mean age of occurrence of mature teratomas in this study was 31 years (SD12.3) this is similar to findings of other studies that suggest that they usually occur in children and young adults [11, 12]. 72.2% of the malignant germ cell tumours occurred in patients younger than 24 years, a finding that is in agreement with the observation that the younger the patient the more likely it is that the germ cell tumour will be malignant [11, 12]. However unlike a study in Ibadan, Nigeria in which 34.5% of germ cell tumours in children up to 14 years were malignant, in this study, only 23.1% of germ cell tumours in this age group were malignant. Instead, 76.9% of malignant germ cell tumours occurred in the 15–24years age range, older than the age group of the subjects in the Ibadan study [8]. The sample size in this study was however larger than the Ibadan study. The mean age of patients with the 46 adult granulosa cell tumours (the commonest sex cord-stromal tumour) was 46.5 years (SD15.9), lower than the 52 years reported in the United States of America [18].

Most tumours in children and adolescents were germ cell tumours; 44 (65.7%), dominated by mature teratomas (53.7%). These were followed by sex cord-stromal tumours 10 (14.9%) and then by Burkitt lymphoma which was the single most common malignant tumour in children and adolescents (30%) and were all bilateral. 2 occurred in children 5–9 years old and 4 in adolescents 10–14 years old, similar to other studies in the West African sub-region [9, 10, 20] and supports the observation that there are a higher proportion of children presenting with malignant ovarian tumours in the West African sub-region as compared to other studies conducted in areas where Burkitt lymphoma is not common [9, 10]. Germ cell tumours as a group however still contributed a larger proportion of tumours in this age group.

### Clinical features

Most of the patients (38.5%) in this study complained of a mass in the lower abdomen unlike patients in a study by Khan and Sultana in Pakistan, in which 57.3% of patients complained of abdominal pain [21]. Concerning duration of symptoms and signs 47.7% had symptoms for less than 1 month while 42.4% had symptoms for between 1 month and 1 year. This differs from the study in Pakistan, where the median duration of symptoms before interview or diagnosis was 12 months [21]. The symptoms encountered in that study that were also present in this study includ abdominal pain, and abdominal distension. The varying clinical presentations of patients with ovarian tumour suggest that there no specific signs or symptoms indicative of ovarian tumours and thus all persistent symptoms and signs should prompt further investigation to rule out ovarian tumour [11, 21].

As regards the size of ovarian surface epithelial tumours 88.9% of the mucinous tumours were less than 20 cm, with 55.6% of the tumours ranging in sizes 11–20 cm. This is compared to 48% of serous tumours. This supports previous reports that on average mucinous tumours tend to be larger than serous tumours [11]. This may be due to the observation that mucinous tumours are more likely to be unilateral and hence are noticed later when they are larger compared to serous tumours that tend to be bilateral and therefore present earlier, an observation that was also made in this study.

## Conclusion

Mature teratoma was the most common ovarian tumour with most occurring in the young. Malignant surface epithelial tumours were the most common malignant ovarian tumours and presented at an earlier age comparable to African- Americans but at variance with findings of other studies involving Indians and Whites. Patients with mucinous carcinoma were younger (47.3 years) compared to those with serous carcinomas (50.1 years). The most frequently occurring single malignant ovarian tumour in women younger than 20 years in this study was Burkitt lymphoma a tumour unique to our geographical location. Statistically, no symptoms and signs specifically indicate that a patient has a malignant ovarian tumour either in early or late stages to assist with diagnosis.

## Abbreviations

GCT: Granulosa cell tumour
H&E: Haematoxylin and eosin
ID: Intermediate differentiation
KBTH: Korle-bu teaching hospital
PAS: Periodic acid schiff
SD: Standard deviation
